# Thermal Conductivity of Molten Carbonates with Dispersed Solid Oxide from Differential Scanning Calorimetry

**DOI:** 10.3390/ma12091486

**Published:** 2019-05-08

**Authors:** Sathiyaraj Kandhasamy, Anne Støre, Geir Martin Haarberg, Signe Kjelstrup, Asbjørn Solheim

**Affiliations:** 1 Department of Materials Science and Engineering, Norwegian University of Science and Technology (NTNU), NO-7034 Trondheim, Norway; geir.martin.haarberg@ntnu.no; 2 SINTEF Industry, SINTEF, NO-7034 Trondheim, Norway; Anne.Store@sintef.no (A.S.); Asbjorn.Solheim@sintef.no (A.S.); 3 PoreLab, Department of Chemistry, NTNU, NO-7034 Trondheim, Norway; signe.kjelstrup@ntnu.no

**Keywords:** Molten salt composite, thermal conductivity, differential scanning calorimetry, laser flash analysis

## Abstract

Recently, there has been a noticeable increase in the applications of composite mixtures containing molten salt and solid oxide for thermal energy conversion and storage systems. This highlights that thermal conductivity of the composites are central for the purpose of designing and devising processes. Measuring the thermal conductivity of molten samples at elevated temperatures remains challenging. In this study, the possibility to use heat flux differential scanning calorimetry (DSC) to measure the thermal conductivity of molten samples at elevated temperatures is reported for the first time. The thermal conductivity of composite mixtures containing eutectic (Li,Na)_2_CO_3_ with and without selected solid oxides at ~675 °C was determined by using the proposed DSC approach. This mixture is a candidate for high temperature waste heat conversion to electric energy. In the DSC measurement program, steps with repeated thermal cycles between 410 and 515 °C were included to limit the effect of the interface thermal contact resistance. The determined values 0.826 ± 0.001, and 0.077 ± 0.004 W m^−1^K^−1^ for the carbonate mixtures with and without solid MgO were found to match the reliable analysis at similar conditions.

## 1. Introduction

Molten salt based composite mixtures are emerging as vital components for many high temperature heat conversion and storage systems [1,2,3,4]. The latest concentrating solar power (CSP) plants are using such composite mixtures for thermal energy storage (TES) [3,5,6]. Recently, electrolyte mixtures, in the form of composites with molten carbonate and solid oxide, were found to be suitable for high temperature thermoelectric cells with liquid electrolyte (i.e., thermocell) [4,7,8]. Utilization of these composites was found to improve conditions for operation and enhance efficiency. Among physical and chemical properties of the composites, thermal conductivity is a critical factor for designing the process [9,10,11,12]. 

Many high temperature thermal conductivity measurement techniques have been established, such as transient hot-wire/strip and laser flash analysis (LFA) [13,14]. However, due to the high electrical conductivity, the transient hot-wire requires a non-conducting probe to measure in melts [15,16]. The LFA analysis demands a special sample container to measure the molten samples and high sampling precision to form an ideal three-layer interface [17]. These modifications make the analysis expensive and difficult to perform. Differential scanning calorimetry (DSC) is a simple and common tool that can measure several thermal properties, such as heat capacity, melting point, and phase transition temperature [18]. It is suitable for a variety of samples (solids, powders, thin films, and melts). 

In DSC, the heat flow into the sample is monitored, and used to determine the thermal properties. The sensor, integrated with the furnace (bottom of the pan), measures the heat flowing into the sample. Furthermore, in DSC, for measurements under 700 °C error due to heat transfer by radiation is not significant. This is because the furnace used to heat sample in the DSC apparatus is assembled with a sequence of radiation shields (see Figure S1) to avoid heat loss by radiation [19]. However, the heat flowing out from the sample is not measured, but this is also essential for computation of the thermal conductivity of the sample. In 1985, Hakvoort et al. [20] demonstrated the possibility of using the heat flux in DSC to measure thermal conductivity of solids. The heat flowing out from the sample was indirectly determined by placing a pure metal reference over the sample and the melting behavior of the metal was recorded [20]. Thus, the change in melting behavior of the metal revealed the thermal resistance of the sample. 

Hakvoort et al. [20] determined the thermal conductivity of a few solid samples by using a Ga or In metal reference on top of the sample. The solid samples with a thermal conductivity of 1 W m^−1^K^−1^ or higher showed reliable results only when the thermal contact was improved by applying a special paste. But for the solid samples with low thermal conductivity (like plastic or porous materials), application of contact paste was not strictly required provided the samples are sufficiently flat. In the case of porous solid samples, the gas atmosphere used in the analysis was found to influence the estimated thermal conductivity of the samples [20]. Moreover, the value for thermal conductivity of a few powders and polymer films has been determined by DSC at low temperatures [21,22,23,24,25,26]. Recently, Hamidreza et al. [27,28,29,30,31] reported an appropriate approach to deconvolute the thermal contact resistance from the measured total thermal resistance of the sample. For the purpose of accurate thermal conductivity, a two-thickness method (i.e., identical samples with different thickness) was considered and the average value of their actual thermal conductivities was used [27,28,31]. In the present study, for the first time, we optimize experimental conditions to use with heat flux DSC for computation of the thermal conductivity of the molten salt composite mixtures at higher temperatures. The thermal conductivity of a sample is also determined by using LFA analysis for validation of the DSC approach.

## 2. Materials and Methods

The sample compositions are listed in Table 1. High purity (>99%) molten carbonates and solid oxides were purchased from Sigma-Aldrich. Samples were prepared by mixing the as-purchased chemicals in a hand mortar and pestle; then dried in a hot air oven at 200 °C for 48 h. A NETZSCH STA 449C Jupiter apparatus for simultaneous DSC and thermal gravimetric analysis (TGA) was used for the analysis. The DSC metal standards were used to calibrate the apparatus. The dried powders were packed into the DSC cylindrical alumina pan (~5.8 mm inner dia and 3.9 mm inner height) by a gentle press. Following this, (Figure S1) the pure Al metal was machined as a flat surfaced disk (5 mm dia and 1 mm thick) and was slightly pressed over the packed powder sample in the DSC pan. To obtain an accurate thermal resistance of the sample, the heat flow path to the metal should be confined through the sample. To ensure the essential heat flow path, the Al disk was centered over the sample to avoid contact with the pan walls. First, a blank measurement was executed with two identical empty pans placed in the two compartments of the instrument for the background correction. After this, an empty pan was replaced by the pan loaded with the sample and Al disk. The heat flow into the sample was recorded as a function of temperature under N_2_ atmosphere. For each sample, the measurement was performed with three different quantities (30, 45, and 60 mg) of the powder sample. A multi-thickness method was used, where the sample composition and Al reference disk remain the same, with the only difference being the sample thickness. A new pan and Al disk were used for each measurement, the disks were identical, with an equal weight of 52.8 mg (±0.3 error).

The thermal conductivity of an LNC-MO (eutectic (Li,Na)_2_CO_3_ dispersed with solid MgO) sample was also determined by NETZSCH 457 MicroFlash (LFA) for comparison. A special container from NETZSCH made of an alloy (Pt10%Rh) with a top lid was used to measure the sample in the molten phase. The outer surface of the container was coated with a thin layer of graphite to avoid laser reflection. To avoid the formation of interface voids (container-sample-top lid) and overflow of the sample on melting, a specific amount of the sample was used to fill the container. A quantity of 2 g of the as-prepared LNC-MO powder was compacted into a circular disk (10 mm diameter) in a hydraulic press with a load of 250 kg. In the LFA instrument, the container with a LNC-MO disk was heated to the measurement temperature under N_2_ atmosphere. Once the measurement temperature (675 °C) was established, a short laser beam heated one side of the sample. The temperature rise on the other side was measured by the infrared detector (IR) detector to estimate thermal diffusivity (α). The sample was exposed to 5 successive laser shots for better accuracy, with an interval of 1 min between each subsequent shot. The measurement was repeated with a new LNC-MO disk to confirm the reproducibility. The measured thermal diffusivity (α) was converted into thermal conductivity (λ) through the expression λ = αρC_p_ [32]. A push rod dilatometer was used to determine the density (ρ) and a separate DSC measurement (Table S1) was performed to attain the heat capacity (C_p_). The procedure to determine the ρ and C_p_ of the samples is provided in the Supplementary material (Figures S2 and S3). 

## 3. Results and Discussion

**Figure 1:**
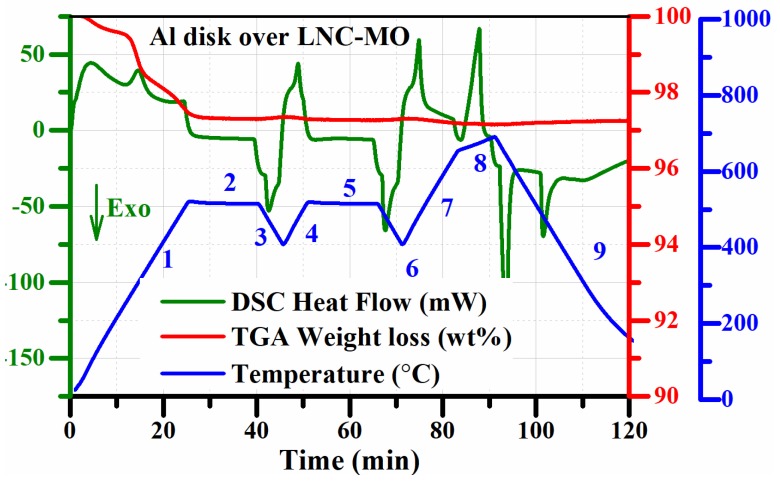
Recorded DSC heat flow and TGA weight loss profiles (numbers along the temperature profile are the segments listed in Table 2).

**Figure 2:**
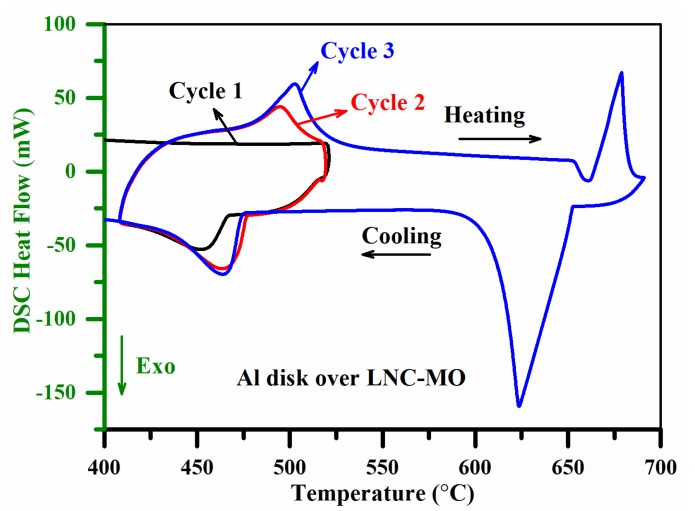
Influence of the thermal cycles and reduction in thermal contact resistance.

The composition of the samples (Table 1) under investigation were used as candidates for high temperature thermal energy conversion and storage systems. For thermoelectric energy conversion and thermal energy storage operations, it is essential to know the solid–liquid phase transition of the salt in the composite mixture. The systems are always operated at temperatures well above the melting point of the molten salt. Therefore, the DSC thermal conductivity measurement program (Table 2) was set up, considering the melting point of the molten salt mixtures along with a few other parameters, such as the thermal and chemical stability of the samples and the melting point of the metal disk. In Figure 1, the TGA weight loss detected before the first heating cycle was due to evaporation of moisture (<200 °C) and melting (~495 °C) of LNC (eutectic Li and Na carbonates) in the LNC-MO mixture [33]. No significant weight loss was observed, demonstrating a high thermal and chemical stability of the sample during the analysis [34]. The steady baseline of the DSC cooling curves (Figure 2) before the exothermic peaks also confirms the chemical stability and retention of the homogeneity of the sample [33,35]. The LNC in the sample mixtures had a liquidus temperature of ~495 °C [4]. Therefore, the selection of pure Al metal (melting point ~660 °C) as the reference is reasonable.

The interface thermal contact resistance between the pan, sample, and Al disk could add an additional thermal resistance. In case of solid samples, Hakvoort et al. [20] reported that applying a silicon paste with high thermal conductivity at the interface minimized the effect of contact resistance. The silicon paste at the interface could, however, also affect the homogeneity of the sample in the molten state. It is well-known that the LNC in the sample mixture undergoes a significant thermal expansion and contraction upon heating and cooling, with a liquidus temperature of ~495 °C [33,36]. Therefore, subjecting the sample to an appropriate thermal cycling (heating and cooling) could improve the thermal contact. Thus, steps with repeated thermal cycles between 410 and 515 °C were included to limit the effect of the interface thermal contact resistance between the pan, sample, and Al disk. Meanwhile, in this DSC approach, the heating curve between 650 and 690 °C showing the melting behavior of the Al disk, is the only essential region to determine the thermal conductivity of the sample. Therefore, the Al disk should melt with a sharp endothermic peak to display the change in heat flowing out from the sample [21,22]. To hold the Al disk in its solid phase during the thermal cycles, the heating temperature of the thermal cycles was limited to 515 °C. However, the isothermal step for 15 min at 515 °C will melt LNC completely while establishing the solid–liquid phase transition. A slow heating rate (5 °C/min) was used to execute the particular region (650 to 690 °C) showing the Al melting behavior. Otherwise, a relatively fast rate (20 °C/min) was used to shorten the analysis time. 

**Figure 3:**
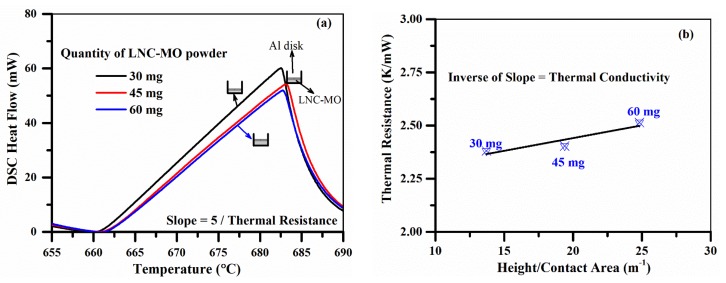
(**a**) The endothermic peak (segment 8—Al disk melting) of measurements with different quantity of LNC-MO, (**b**) The plot is used to estimate the absolute thermal conductivity.

The DSC endothermic peak (Figure 2) observed in the second and third heating cycles represents the LNC solid to liquid phase transition. The endothermic peak is intense and well defined in the third heating cycle, but not even initiated in the first heating cycle. This illustrates that the repeated solid and liquid phase transition had established a better thermal contact after a few thermal cycles [33,34]. The heating curve recorded between 650 and 690 °C (Al melting behavior) in the third cycle (after establishing a better thermal contact) was used to determine the thermal resistance of the sample. Hence, further deconvolution of thermal contact resistance from the estimated bulk resistance was not considered. Figure 3a shows that the Al melting point was not at the exact expected temperature of 660 °C. This shift may be associated with the thermal resistance of the heat flow path (sample) [20,21]. However, Al melting behavior was initiated around 660 °C (±2 °C) in all measurements. The difference in sample quantity will change the heat flow path length (i.e., the sample height) and consequently shift the measured Al melting point (Figure 3a) [21,22]. The thermal resistance of each measurement was determined from the slope of the rising side of the endothermic peak [20,21,22]. The temperature of the measurement was considered to be ~675 °C, the average temperature of the endothermic peak’s rising side.

The calculated thermal resistances were plotted against the corresponding height to contact area ratio of the sample in Figure 3b [21,22]. The contact area between the molten sample and pan will be the same as the inner cross-sectional area of the pan. However, the height (thickness) of the molten sample packed between the pan and the Al disk was varied, and was determined from the quantity and density of the sample mixture. The change in density of the sample on heating was estimated by using the push rod dilatometer (Supplementary material Figure S2) to process the LFA measurement for better accuracy. The estimated density at 675 °C, and the known sample quantity, were used to determine the appropriate change in sample height. The appropriate height (thickness) for the different quantities (30, 45, and 60 mg) of the molten LNC-MO sample was determined to be 0.36, 0.51, and 0.66 mm. Therefore, a multi-thickness method was used, where the sample composition and Al reference disk remain same with only difference in sample thickness. The inverse of the slope of the linear fit (Figure 3b) is the absolute thermal conductivity of the LNC-MO (0.08 ± 0.004 W m^−1^K^−1^) [22]. The slope of the linear fit and associated standard error were determined by linear least-square regression analysis. Therefore, average of actual thermal conductivity of the measurements with different thickness of same sample was reported as measured thermal conductivity in Table 3. In a similar way, the thermal conductivity (Table 3) of the other samples was also determined using the DSC method (Figures S4 and S5).

Table 3 shows that the thermal conductivity of the LNC-MO mixture determined by the DSC (0.077 ± 0.004 W m**^−^**^1^K**^−^**^1^) and the LFA (0.078 ± 0.001 W m**^−^**^1^K**^−^**^1^) was the same within the ascertained accuracy of the experiment. The LFA thermal diffusivity measured for the five successive laser shots were converted into thermal conductivity individually, as shown in Table 4. The absolute value and standard deviation reported from the LFA analysis in Table 3 is mean and standard error of the mean of the thermal conductivity for the five shots in Table 4. While the LFA technique requires a special container and separate specific heat capacity (C_p_) measurement for data processing, the DSC method is less expensive. Thus, the thermal conductivity measurement by the DSC method is inexpensive compared to the LFA method. We have seen that the thermal conductivity of the pure eutectic (Li,Na)_2_CO_3_ melt (i.e., LNC), determined by using the DSC approach (0.83 ± 0.001 W m**^−^**^1^K**^−^**^1^), matches with a reliable data set (0.887 ± 0.002 W m**^−^**^1^K**^−^**^1^) [15].

## 4. Conclusions

The thermal conductivity of composite mixtures containing molten carbonate and solid oxides was determined by the DSC approach. For the mixtures containing eutectic (Li,Na)_2_CO_3_ with and without solid MgO, the measured thermal conductivity was identical to the LFA and a reliable data set in the literature [15]. The DSC approach appears to be a simple and inexpensive method to determine the thermal conductivity of molten samples. Therefore, the heat flux DSC analysis is a suitable tool to determine the thermal conductivity of molten salt composites at elevated temperatures. We reported new data for thermal conductivity of eutectic (Li,Na)_2_CO_3_ with and without dispersed solid oxide (MgO / LiAl_2_O_3_ / Al_2_O_3_ / CeO_2_) at 675 °C.

## Figures and Tables

**Table 1:** The composition of the samples used in the experiments.

Sample	Sample Composition		Composition Ratio (vol %)	
	Molten Salt	Solid Oxide	Molten Salt	Solid Oxide
LNC-MO	Eutectic molten carbonate (0.53 mol Li_2_CO_3_ + 0.47 mol Na_2_CO_3)_	MgO	45	55
LNC-AO		Al_2_O_3_	45	55
LNC-CO		CeO_2_	45	55
LNC-LAO		LiAl_2_O_3_	45	55
LNC		--	100	--

**Table 2:** The differential scanning calorimetry (DSC) thermal conductivity measurement program.

Segment	Mode	Temperature (°C)	Rate (°C/min)	Hold Time (min)
1	Dynamic (Heating)	30–515	20	---
2	Isothermal	515	---	15
3	Dynamic (Cooling)	515–410	20	---
4	Dynamic (Heating)	410–515	20	---
5	Isothermal	515	---	15
6	Dynamic (Cooling)	515–410	20	---
7	Dynamic (Heating)	410–650	20	---
8	Dynamic (Heating)	650–690	5	---
9	Dynamic (Cooling)	690–30	20	---

**Table 3:** The estimated thermal conductivity of the samples.

Sample	Thermal Conductivity (W m^−1^K^−1^)		
	DSC	LFA	Literature [15]
LNC-MO	0.077 ± 0.004	0.078 ± 0.001	--
LNC-AO	0.039 ± 0.006	--	--
LNC-CO	0.038 ± 0.004	--	--
LNC-LAO	0.066 ± 0.011	--	--
LNC	0.826 ± 0.001	--	0.887 ± 0.002

**Table 4:** The laser flash analysis (LFA) thermal diffusivity and estimated thermal conductivity of LNC-MO at 675 °C.

Laser Shot Number	Thermal Diffusivity (mm^2^/s)	Thermal Conductivity (W m^−1^K^−1^)
1	0.301	0.078
2	0.300	0.078
3	0.297	0.077
4	0.298	0.077
5	0.304	0.079

## Supplementary Materials

The following are available online at https://www.mdpi.com/1996-1944/12/9/1486/s1, Full experimental procedure used to determine the density and heat capacity of the samples; Figure S1: (a) STA 449 C Jupiter^®^ equipment, (b) Schematic of NETZCH STA 449C Jupiter and, (c) LNC-MO sample mixture, Al metal disk and DSC alumina crucible; Figure S2: Dilatometer measurement to determine the change in density; Figure S3: DSC measurement to determine the Cp of LNC-MO; Figure S4 and S5: Recorded DSC heat flow and TGA weight loss profiles of LNC-AO, LNC-CO, LNC-LAO and LNC samples (30 mg of sample); Table S1: DSC temperature program used in determining the Cp.
