# Supplementary material for: Thermal Conductivity of Molten Carbonates with Dispersed Solid Oxide from Differential Scanning Calorimetry
Source: Materials (Basel). 2019 May 8;12(9):1486. doi: 10.3390/ma12091486 (PMC6539058; doi:10.3390/ma12091486)
Supplement: Supplementary file 1 [file materials-12-01486-s001.pdf]

Supporting Information
Thermal Conductivity of Molten Carbonates with Dispersed Solid Oxide from Differential Scanning Calorimetry

Sathiyaraj Kandhasamy [1,*], Anne Støre [2], Geir Martin Haarberg [1], Signe Kjelstrup [3] and Asbjørn Solheim [2]

1 Department of Materials Science and Engineering, Norwegian University of Science and Technology (NTNU), NO-7034 Trondheim, Norway; geir.martin.haarberg@ntnu.no (G.M.H.)
2 SINTEF Industry, SINTEF, NO-7034 Trondheim, Norway; Anne.Store@sintef.no (A.S.); Asbjorn.Solheim@sintef.no (A.S.)
3 PoreLab, Department of Chemistry, NTNU, NO-7034 Trondheim, Norway; signe.kjelstrup@ntnu.no
* Correspondence: k.sathiyaraj14@gmail.com or sathiyaraj.kandhasamy@ntnu.no

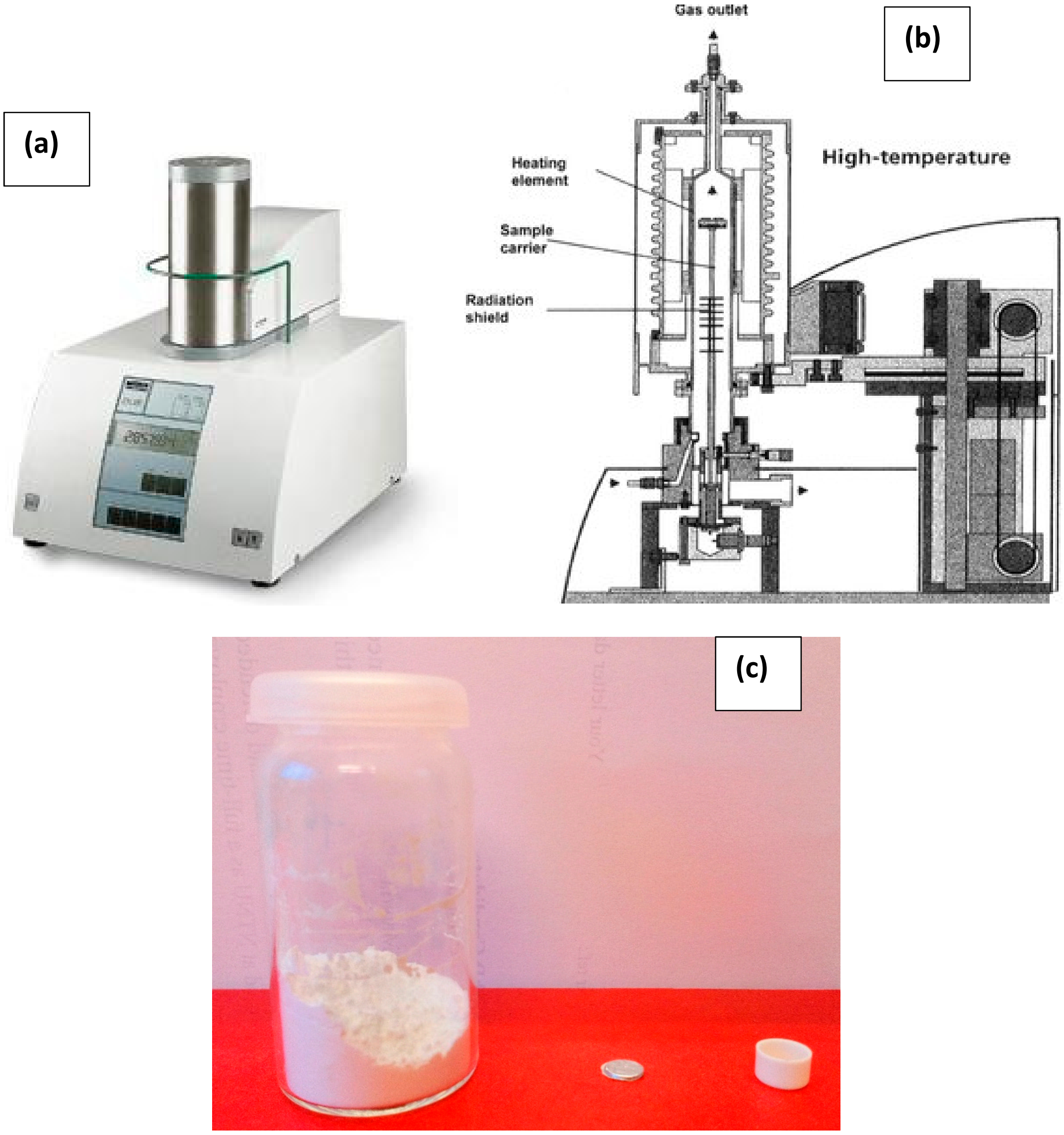

Figure S1. (**a**) STA 449 C Jupiter® equipment, (**b**) Schematic of NETZCH STA 449C Jupiter and, (**c**) LNC-MO sample mixture, Al metal disk and DSC alumina crucible.

Change in density (ϱ)

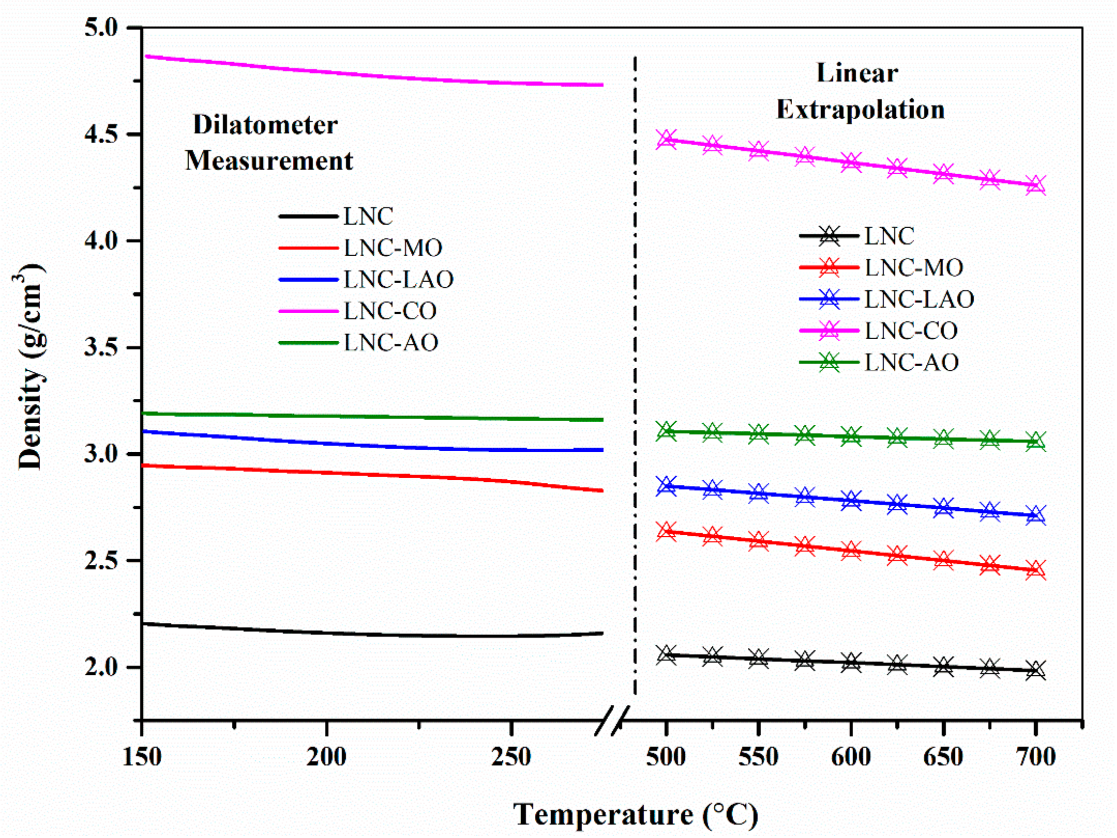

Figure S2. Dilatometer measurement to determine the change in density.

The thermal expansion of the samples on heating is determined by using a pushrod dilatometer DIL 402 C from Netzsch. For dilatometer analysis, a cylindrically shaped pellet is prepared in 5 mm diameter with ~0.25 g of the sample powder by applying a load of 250 kg in a hydraulic press. This pushrod dilatometer is equipped for the solid samples, so the maximum measurement temperature is limited to 300 °C (sample melts at ~495 °C). A standard alumina sample is used in blank measurement for background correction. A slow heating rate (2 K/min) was used for both heating and cooling the samples, for measurement accuracy. Both the measurements of the samples and blank were performed under the nitrogen atmosphere. The measured thermal expansion is used to calculate the change in density in the solid phase (Figure S2). The change in density of the samples in the solid phase is used to extrapolate the density at the higher temperature for molten phase. The extrapolated density shows a decreasing tendency by increasing temperature, identical to the literature with binary molten carbonates. Even though the density of LNC-MO with 55 vol% of solid oxide (MgO) varies slightly, the trend of change in density is similar to the LNC.

Heat Capacity (Cp)

Table S1. DSC temperature program used in determining the C_P.

Segment	Mode	Temperature (°C)	Rate (°C/min)	Hold Time (min)
1	Dynamic (Heating)	30–515	20	---
2	Isothermal	515	---	15
3	Dynamic (Cooling)	515–410	20	---
4	Dynamic (Heating)	410–515	20	---
5	Isothermal	515	---	15
6	Dynamic (Cooling)	515–410	20	---
7	Dynamic (Heating)	410–470	20	---
8	Isothermal	470	---	30
9	Dynamic (Heating)	470–700	5	---
10	Dynamic (Cooling)	690–30	20	---

Table S1 shows the DSC measurement program used to determine the C_P. Here, the Al disks are not placed over the sample, but the experimental procedure was similar as for the DSC thermal conductivity measurement. The heat flow into the sample was recorded as a function of temperature under N_2 atmosphere. In Figure S3, the heating curve after the isothermal step is used for the direct measurement of heat capacity (C_P). An isothermal step at 470 °C (segment 8) and a slow heating rate between 470 and 700 °C (segment 9) was included to establish a stable temperature equilibrium in the sample. These modifications in the DSC measurement program for C_P measurement (Figure S3) leads to a longer analysis time compared to the DSC thermal conductivity measurement shown in Figure 1.

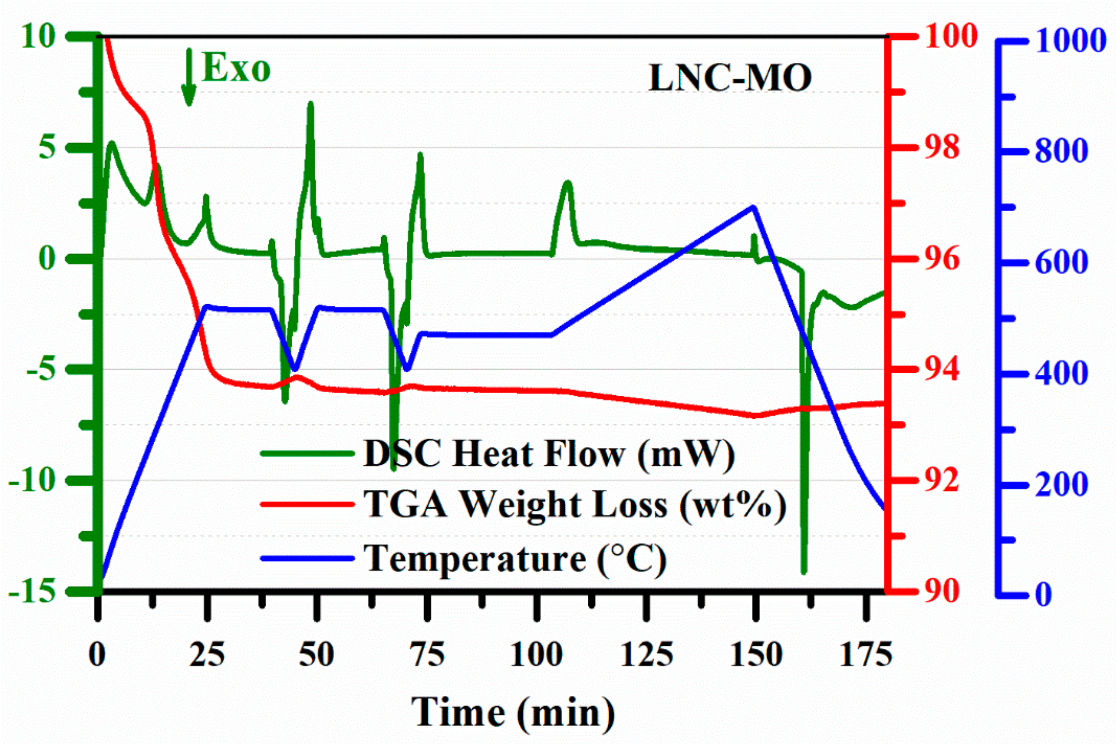

Figure S3. DSC measurement to determine the C_P of LNC-MO.

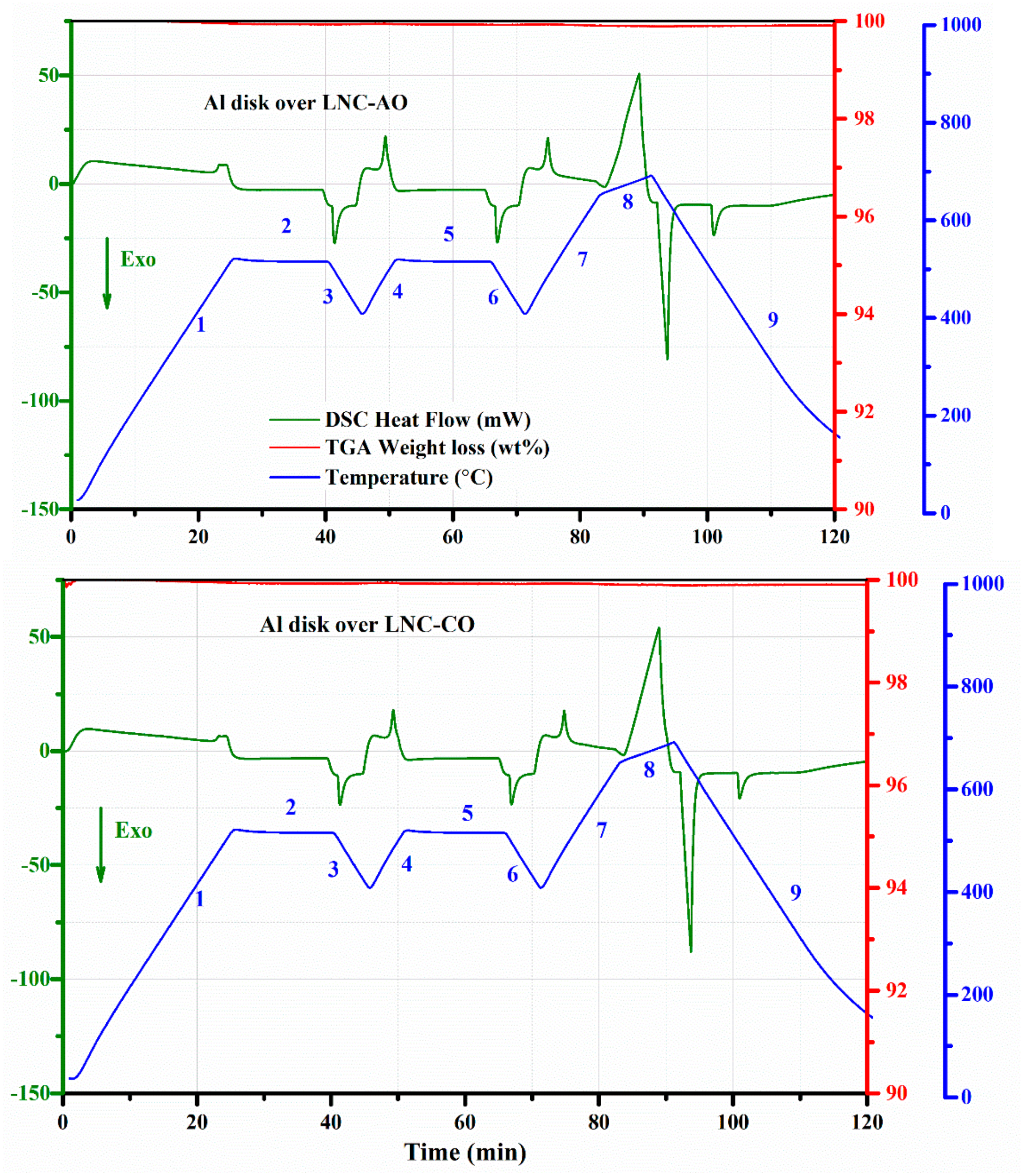

Figure S4. Recorded differential scanning calorimetry (DSC) heat flow and TGA weight loss profiles of LNC-AO and LNC-CO samples (30 mg of sample).

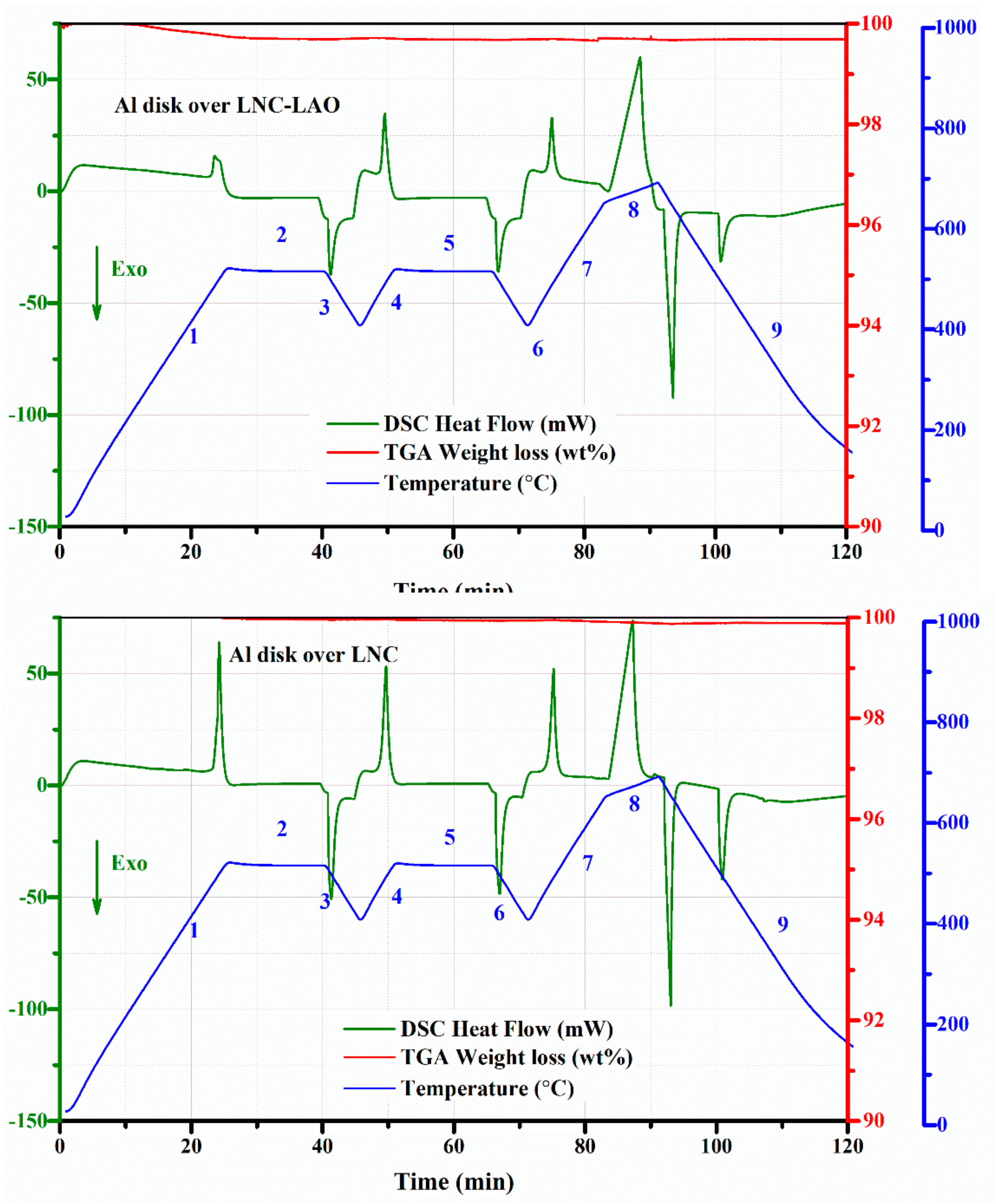

Figure S5. Recorded DSC heat flow and TGA weight loss profiles of LNC-LAO and LNC samples (30 mg of sample).

The laser flash analysis (LFA) analysis only measures the thermal diffusivity (α) of the sample. Usually an expression $\lambda = \alpha \varrho C_p$ is used to convert the measured thermal diffusivity (α) into thermal conductivity (λ). This requires the density (ϱ) and heat capacity (C_p) of the samples at the particular temperature. A push rod dilatometer was used to determine the ϱ and a separate differential scanning calorimetry (DSC) measurement was performed to attain the C_p.
